# Melatonin-Mediated Enhancement of Photosynthetic Capacity and Photoprotection Improves Salt Tolerance in Wheat

**DOI:** 10.3390/plants12233984

**Published:** 2023-11-27

**Authors:** Di Yan, Jiajie Wang, Zhenzong Lu, Rui Liu, Yue Hong, Baocai Su, Ye Wang, Zhen Peng, Chunxin Yu, Yuerong Gao, Ziyan Liu, Zhaoshi Xu, Liusheng Duan, Runzhi Li

**Affiliations:** 1 College of Plant Science and Technology, Beijing University of Agriculture, Beijing 102206, China; di.yan@bua.edu.cn (D.Y.); jiajie.wang@bua.edu.cn (J.W.); zhenzong.lu@bua.edu.cn (Z.L.); rui.liu@bua.edu.cn (R.L.); yue.hong@bua.edu.cn (Y.H.); baocai.su@bua.edu.cn (B.S.); ye.wang@bua.edu.cn (Y.W.); zhen.peng@bua.edu.cn (Z.P.); chunxin.yu@bua.edu.cn (C.Y.); yuerong.gao@bua.edu.cn (Y.G.); ziyan.liu@bua.edu.cn (Z.L.); duanlsh@cau.edu.cn (L.D.); 2 Beijing Key Laboratory for Agricultural Application and New Technique, Beijing 102206, China; 3 Institute of Crop Sciences, Chinese Academy of Agricultural Sciences, Beijing 100081, China; xuzhaoshi@caas.cn; 4 College of Agronomy and Biotechnology, China Agricultural University, Beijing 100193, China

**Keywords:** wheat (*Triticum aestivum* L), salt stress, melatonin, RNA-seq, photosynthesis

## Abstract

The role of melatonin in plant growth and response to environmental stress has been widely demonstrated. However, the physiological and molecular regulation of salt tolerance in wheat seedlings by melatonin remains unclear. In this study, we investigated changes in phenotype, physiology, photosynthetic parameters, and transcript levels in wheat seedlings to reveal the role of melatonin in the regulation of salt tolerance in wheat. The results indicate that the application of exogenous melatonin significantly alleviates growth inhibition, reactive oxygen species accumulation, and membrane oxidative damage induced by salt stress in wheat. Additionally, exogenous melatonin increased antioxidant enzyme activity and regulated photosynthetic gas exchange. Transcriptomic data showed a significant up-regulation of genes encoding light-harvesting chlorophyll protein complex proteins in photosynthesis and genes related to chlorophyll and carotenoid biosynthesis under the influence of melatonin. These results suggest that exogenous melatonin improves salt tolerance in wheat seedlings by enhancing the antioxidant, photoprotective, and photosynthesis activities.

## 1. Introduction

Soil salinization is a global issue resulting from both global climate change and inappropriate agricultural production, including incorrect farming techniques and excessive fertilizer application [1,2]. More than 20% of the world’s arable land has been threatened by salt stress, which is severely reducing food production [3,4], threatening sustainable agricultural development and global food security [5,6,7,8]. Salt stress causes osmotic stress, ionic toxicity, and oxidative damage to plants, which in turn inhibits seed germination, development, flowering and fruiting, and ultimately leads to reduced crop yields [9,10,11,12]. Salt-mediated osmotic stress reduces the ability of plants to draw nutrients and water from the soil, leading to growth stagnation [1,13]. Ionic toxicity caused by high Na^+^ and Cl^−^ concentrations disrupts cellular homeostasis, increases membrane peroxidation, and inhibits photosynthesis in plants [14,15]. In addition, salt stress induces the accumulation of reactive oxygen species (ROS), such as hydrogen peroxide (H_2_O_2_) and superoxide anion (O_2_^−^), resulting in oxidative damage and inhibition of plant growth and development [16,17].

Plants have developed a complex mechanism against salt stress, including regulating endogenous hormone levels, ion homeostasis, osmotic potential, and activity of antioxidant enzymes [18,19,20]. Plants control ion uptake and transport by the salt overly sensitive (SOS) signaling pathway, which can maintain Na^+^/K^+^ in cells by exporting excess Na^+^ when triggered by Ca^2+^ in the cytoplasm [21,22,23,24,25]. Moreover, plants remove excess Na^+^ via Na^+^/H^+^ antiporters located at plasma and vesicle membranes [17,26,27]. Plant cells can increase the osmotic potential by accumulating osmoregulatory substances, such as soluble sugars, proline, inorganic ions, betaine, and polyols, thereby increasing salt tolerance [28]. ROS, which play an essential role in cell signaling, will be increased dramatically in plants when subjected to adversity stress and cause oxidative damage to cellular structures [9,29]. The ROS scavenging system mediated by antioxidant enzymes, including ascorbate peroxidase (APX), superoxide dismutase (SOD), peroxidase (POD), and catalase (CAT), can reduce the extent of cellular damage by scavenging the excessive accumulation of ROS, which is an important way to reduce oxidative damage in plants [7,30].

Melatonin (N-acetyl-5-methoxy tryptamine, MT) is a famous animal hormone involved in many bio-processes that include sleep, mood, day–night rhythms, seasonal reproductive physiology, temperature homeostasis, sexual behavior, antioxidant activity, and immune enhancement [31,32]. However, it is not a hormone unique to animals but is commonly found in plants [33]. Since 1995, when three laboratories simultaneously reported MT in plants, studies have detected and quantified MT in roots, shoots, leaves, fruits, and seeds of a considerable variety of plants [34,35,36]. To this day, MT is understood as a universal regulator of plant growth and development processes and various stress responses [31,37]. For example, MT maintains Na^+^/K^+^ homeostasis under salt stress by increasing root H^+^-pump activity and the sensitivity of Na^+^/K^+^ transporter proteins to ROS and reactive nitrogen species (RNS) [38]. MT has been reported to increase seed germination and the root/stem ratio in cucumber under water stress [39]. Furthermore, MT was reported to reduce chlorophyll degradation, delay leaf senescence, improve antioxidant enzyme activity, increase osmoregulatory substances, and maintain hydrogen peroxide homeostasis in plants under abiotic stress [19,40,41,42]. Melatonin increased chlorophyll content, assimilation rate (A), stomatal conductance (gs), Internal CO_2_ (Ci), and transpiration rate (Tr) and also increased photosystem II (PSII) activity, PSII response center protein D1, Lhcb1, and Lhcb2 in rice, maize, and tomato under various stresses [43,44,45].

Wheat (*Triticum aestivum* L.), the second largest food crop in the world, suffers from the salinization of arable land, which seriously affects yields [4]. Therefore, how to improve the salt tolerance of wheat is gradually becoming an agricultural research hotspot. Although some studies have demonstrated the protective effect of applied exogenous melatonin on plants under salt stress conditions, MT-mediated salinity tolerance mechanisms in wheat seedlings have been studied less. Therefore, it is important to investigate the physiological and molecular mechanisms of wheat seedlings impacted by MT under salt stress, particularly the influence of melatonin on wheat seedling photosynthesis. In this study, wheat biomass, antioxidant enzyme activity, photosynthesis, and transcriptome sequencing were measured for different treatments. The results show that exogenous MT was involved in redox metabolism, alteration of photosynthetic parameters, and biosynthesis of photosynthetic proteins during wheat growth under salt stress, which improved the salt tolerance of wheat at the seedling stage.

## 2. Results

### 2.1. Phenotypes of Wheat Seedlings in Different Treatments under Salt Stress

**Figure 1:**
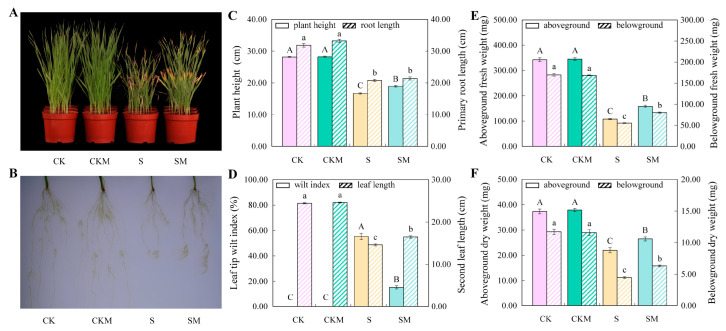
The effect of different conditions (CK, CKM, S, and SM) on the growth of wheat seedlings. (**A**) Phenotypes; (**B**) primary root growth; (**C**) plant height and root length; (**D**) leaf tip wilt index and second leaf length; (**E**) above- and belowground fresh weight; (**F**) dry weight. Values are the averages of replicates ± deviation (SD, n = 3). Different letters indicate significant differences according to the Duncan test (*p* < 0.05). Uppercase letters indicate comparisons between indicators on the left and lowercase letters indicate comparisons between indicators on the right.

After 20 days of growth under salt stress conditions, the phenotypes of wheat seedlings had significant differences (Figure 1). Compared to the seedlings grown under CK (normal condition without MT) and CKM (normal condition with MT) conditions, the plant height and root length of seedlings of S (salt stress without MT) and SM (salt stress whit MT) groups were significantly decreased, and the wilting and yellowing of the first leaves were more serious. After MT application, the plant height and leaf length of SM treatment seedlings significantly increased by 13.66% and 12.70%, respectively, compared to those of untreated seedlings under salt stress, while the leaf tip wilt index significantly decreased by 72.53% (Figure 1A–D). Meanwhile, the application of exogenous MT led to a 46.2% and 47.97% increase in fresh weight and a 20.3% and 45.91% increase in dry weight of aboveground and belowground seedlings, respectively, under salt stress (Figure 1E,F). Notedly, MT had no significant effect on primary root length (Figure 1C), but significantly increased the length and number of lateral roots (Figure 1B). Overall, our results show that melatonin improved the growth of wheat under salt stress but had no significant effect on wheat growth under normal conditions.

### 2.2. Measuring the Antioxidant and Osmoregulatory Abilities of MT

**Figure 2:**
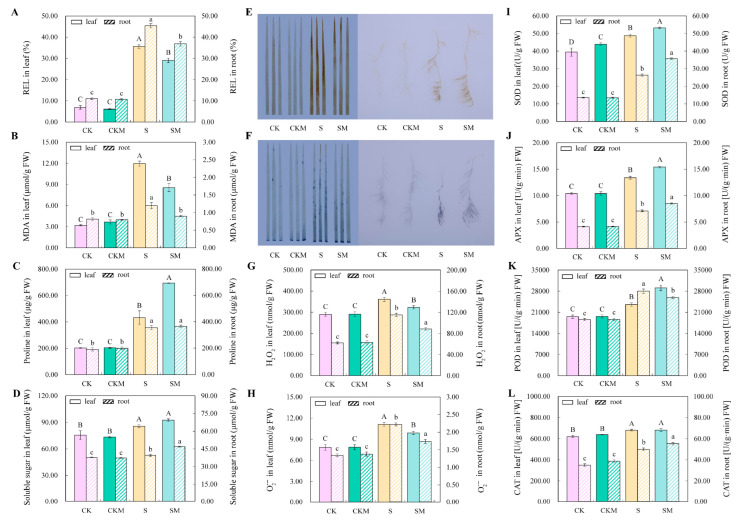
Effect of exogenous MT on physiological indicators of wheat seedlings under salt stress. (**A**) The rate of REL in leaves and roots; (**B**) MDA content in leaves and roots; (**C**) proline content in leaves and roots; (**D**) soluble sugar content in leaves and roots; (**E**) histochemical staining of H_2_O_2_ levels in leaves and roots––H_2_O_2_ was brown-spotted; (**F**) histochemical staining of O_2_^−^ levels in leaves and roots––O_2_^−^ was blue spotted; (**G**) H_2_O_2_ content in leaves and roots; (**H**) O_2_^−^ content in leaves and roots; (**I**) SOD activities in leaves and roots; (**J**) APX activities in leaves and roots; (**K**) POD activities in leaves and roots; (**L**) CAT activities in leaves and roots. Different letters indicate significant differences according to the Duncan test (*p* < 0.05). Uppercase letters indicate comparisons between indicators on the left and lowercase letters indicate comparisons between indicators on the right.

To reveal the antioxidant capacity of MT and its effect on osmoregulation, we determined the REL, MDA content, osmoregulatory substance content, ROS content, and antioxidant enzyme activities of wheat seedlings under different treatments. The results of measuring REL, MDA, proline, and soluble sugar contents of wheat seedlings show a significant increase in both leaves and roots under salt stress, indicating that salt stress damaged the membrane structure of wheat seedlings and induced the accumulation of osmoregulatory substances (Figure 2A–D). However, exogenous MT significantly reduced REL and MDA content in seedlings, while increased proline content in leaves and soluble sugar content in the root system under salt stress were detected, but no significant effect on seedlings under normal conditions (Figure 2A–D), which suggested that MT reduced salt stress-induced oxidative damage and alleviated osmotic stress by increasing osmoregulatory substances.

Under stress conditions, the level of ROS in plants increases dramatically [46]. In this study, exogenous MT had no significant effect on H_2_O_2_ and O_2_^−^ in leaves and roots under normal conditions, but the size and number of brown and blue spots on the leaves and roots were significantly reduced compared to those in untreated seedlings under salt stress, and the results of H_2_O_2_ and O_2_^−^ content measurements were consistent with the staining results (Figure 2E–H). These results indicate that MT can effectively reduce the accumulation of ROS in wheat seedling tissues under salt stress.

The results of antioxidant enzyme (SOD, APX, POD, and CAT) activities assay show that the antioxidant enzyme activities of wheat seedlings applied with exogenous MT did not change significantly under normal conditions, but increased significantly under salt stress (Figure 2I–L). The activities of SOD, APX, and POD in leaves and the activities of SOD, APX, and CAT in the root system were significantly increased (Figure 2I–L).

### 2.3. Photosynthetic Parameters and Chlorophyll Content of Wheat Seedlings under Different Treatments

**Figure 3:**
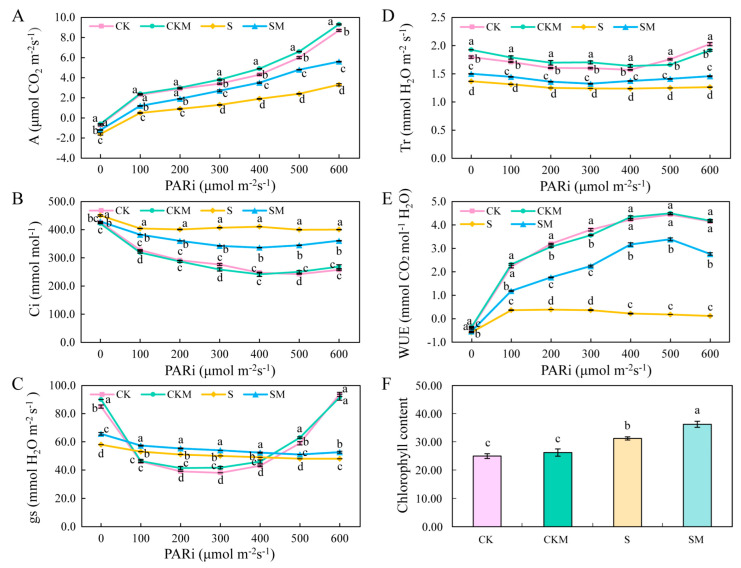
Effect of exogenous MT on photosynthetic parameters of wheat seedlings. (**A**) Assimilation rate (**A**); (**B**) internal CO_2_ (Ci); (**C**) stomatal Conductance (gs); (**D**) transmission rate (Tr); (**E**) water use efficiency (WUE); (**F**) chlorophyll content. Different letters indicate significant differences according to the Duncan test (*p* < 0.05).

The assimilation rate of seedlings was significantly decreased under salt stress compared to normal conditions (Figure 3A). However, exogenous melatonin increased the assimilation rate of seedlings, which was significantly increased by 69.70% at 600 μmol·m^2^·s^−1^ in the SM group compared with the S group (Figure 3A). Compared with normal conditions, Ci was increased by salt stress but decreased after the application of MT (Figure 3B). At different light intensities, the gs and Tr of wheat seedlings in the SM group increased by an average of 9.7% and 12.87%, respectively, compared to the S group (Figure 3C,D). The WUE of wheat increased with increasing light intensity (Figure 3E). Salt-stressed plants showed a significant decrease in the WUE of seedlings, while exogenous MT significantly attenuated the effect of salt stress on WUE (Figure 3E). Salt stress increased the chlorophyll content of wheat, while exogenous MT treatment further increased the chlorophyll content of wheat under salt stress (Figure 3F). These results suggest that MT enhanced the photosynthesis of wheat seedlings under salt stress by improving the assimilation rate and WUE, which promoted the seedlings’ growth.

### 2.4. Comprehensive Transcriptome Analysis of S and SM at Different Times

**Figure 4:**
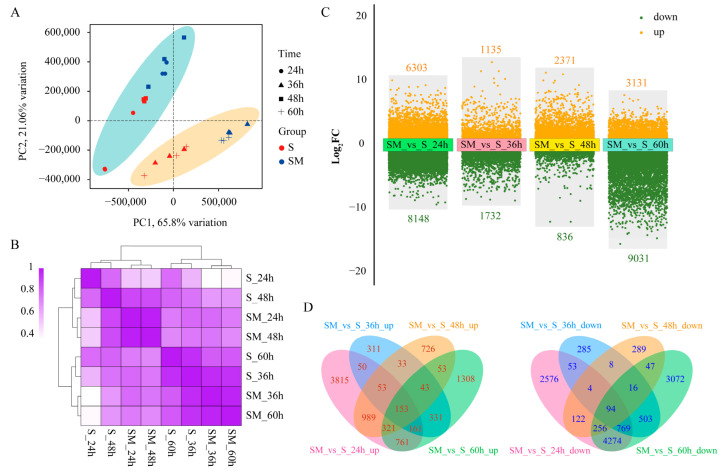
The effect of MT on transcript levels in wheat seedlings from all four different time points under salt stress. (**A**) Principal component analysis (PCA); (**B**) heat map of clustering among treatments; (**C**) DEGs for SM-vs-S at different time points; (**D**) Venn diagram of DEGs.

To investigate the molecular mechanism of exogenous MT to improve salt tolerance in wheat seedlings, we performed RNA-seq on the seedlings of the S group and SM group at 24, 36, 48, and 60 h after MT application. Each sample obtained an average of 7.85 GB of data, with an average of Q30 > 91.26% (Table S2). To verify the reliability of RNA-seq, six genes were randomly selected for qRT-PCR analysis, and the results show that their expression patterns were consistent with the RNA-seq data (Figure S1), indicating that the RNA-seq data are reliable and can be used for further analysis. The results of principal component analysis (PCA) and cluster analysis show that samples from different time points of the same treatment were clustered together, while samples from different treatments had significant dispersion, indicating that MT had a significant effect on transcript levels in seedlings from all four different time points under salt stress (Figure 4A,B).

A total of 14,451 (6303 up-regulated, 8148 down-regulated), 2867 (1135 up-regulated, 1732 down-regulated), 3207 (2371 up-regulated, 836 down-regulated), and 12,162 (3131 up-regulated, 9031 down-regulated) differentially expressed genes (DEGs) were identified in SM treatments compared to S treatments at 24, 36, 48, and 60 h, respectively (Figure 4C). The Venn diagram showed that a total of 153 up-regulated overlapping DEGs and 94 down-regulated overlapping DEGs were identified at all different time points, indicating that these genes were involved in the MT-mediated regulation of salt tolerance in wheat seedlings both day and night (Figure 4D).

### 2.5. Functional and Pathway Annotation of DEGs

**Figure 5:**
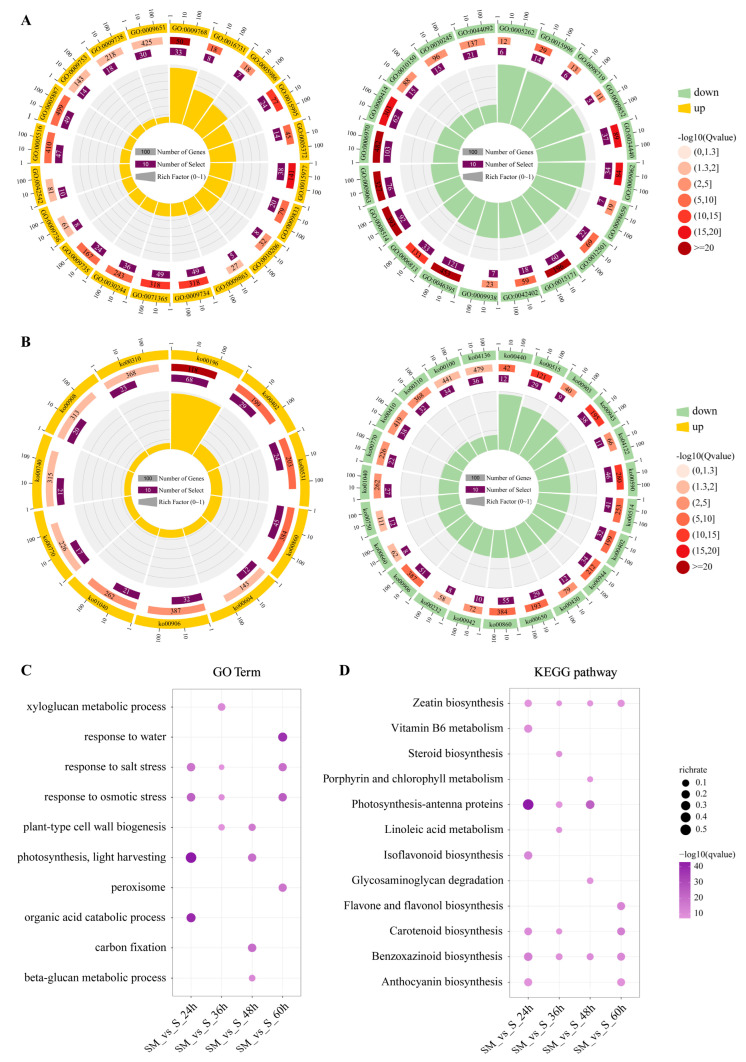
Functional and pathway annotation of DEGs in SM-vs-S comparison. (**A**) Gene ontology (GO) enrichment analysis of DEGs; (**B**) Kyoto Encyclopedia of Genes and Genomes (KEGG) enrichment analysis of DEGs; (**C**) GO enrichment analysis; (**D**) KEGG enrichment analysis of DEGs in different time comparison groups of wheat seedling leaves SM-vs-S.

GO enrichment analysis was performed to annotate the DEGs of seedlings between SM-vs-S comparisons (Figure 5A, Tables S3 and S4). The results show that the up-regulated DEGs were mainly enriched in “photosynthesis, light harvesting in photosystem I (GO:0009768)”, “sucrose biosynthetic process (GO:0005986)”, “response to cytokinin (GO:0009735)”, “chlorophyll biosynthetic process (GO:0015995)”, and “carbon fixation (GO:0015977)” (Figure 5A, Table S3). The down-regulated DEGs in GO terms were mainly enriched in the “chlorophyll catabolic process (GO:0015996)”, “lipid oxidation (GO:0034440)”, “auxin catabolic process (GO:0009852)”, and “sodium ion import across plasma membrane (GO:0098719)” (Figure 5A, Table S4).

To gain insight into the biological processes affected by exogenous MT, we performed KEGG pathway analysis (Figure 5B, Tables S5 and S6). Among them, the up-regulated DEGs mainly involved “photosynthesis-antenna proteins (ko00196)”, “porphyrin and chlorophyll metabolism (ko00860)”, and “carotenoid biosynthesis (ko00906)” pathways (Figure 5B, Table S5). The down-regulated DEGs were mainly enriched in “porphyrin and chlorophyll metabolism (ko00860)” and “arachidonic acid metabolism (ko00590)” (Figure 5B, Table S6). The results of GO and KEGG reveal that MT enhanced the response of wheat to salt stress mainly by regulating the expression of genes involved in photosynthesis, phytohormone regulation, and energy metabolism.

Then, we analyzed the function and pathway of DEGs identified at different time points, respectively. The GO results show that MT-induced DEGs were mainly enriched in stress-responsive terms like “response to osmotic stress” and “response to salt stress” and in terms related to cell structure like “xyloglucan metabolic process” and “plant-type cell wall biogenesis” at 24 h (Figure 5A). In addition, DEGs were mainly enriched in “carbon fixation” and “photosynthesis, light harvesting” which are related to photosynthesis at 48 h, and in “response to water” and “peroxisome” at 60 h (Figure 5A). The results of KEGG enrichment analysis showed that DEGs were enriched to the “benzoxazinoid biosynthesis” and “zeatin biosynthesis” pathways at all times. Photosynthesis-antenna proteins were enriched at 24, 36, and 48 h. In addition, carotenoid biosynthesis was enriched at 24, 36, and 60 h (Figure 5B). Overall, MT improved salt tolerance in wheat by regulating the expression of genes in photosynthesis and antioxidant-related pathways.

### 2.6. Expression Pattern Clustering of the DEGs

**Figure 6:**
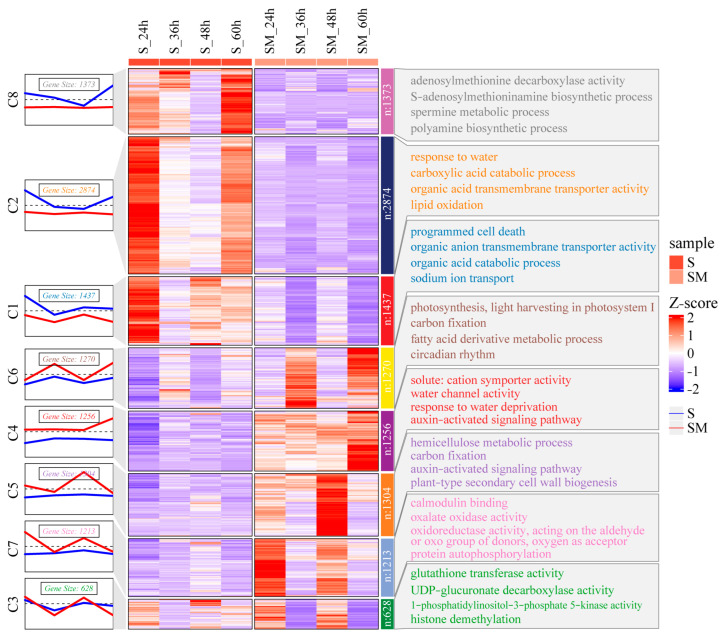
Heat map of expression pattern clustering and GO enrichment analysis of DEGs in SM-vs-S comparison. The folded line graph represented the Mfuzz clustered expression pattern of DEGs.

To investigate the expression pattern of DEGs, we used the Mfuzz clustering method to perform a time series analysis of all DEGs. The result show that all DEGs were clustered into eight clusters according to their different expression patterns (Figure 6). Among them, C1, C2, C4, and C6, which clustered 2874, 1437, 1270, and 1256 DEGs, respectively, were considered to have a great contribution to improved salt tolerance in wheat. The DEGs clustered in C1 and C2 were consistently down-regulated by MT induction, while those clustered in C4 and C6 were consistently up-regulated by MT (Figure 6). GO enrichment analysis showed that the DEGs in C1 were mainly involved in “programmed cell death”, “organic anion transmembrane transporter activity”, and “sodium ion transport”, while the DEGs in C2 were mainly enriched in “response to water”, “carboxylic acid catabolic process”, and “lipid oxidation”. These results suggest that MT had a positive effect on the maintenance of cellular osmotic potential and protected cell structure by inhibiting lipid oxidation and controlling ion transportation. The DEGs in the C6 were mainly associated with “photosynthesis, light harvesting in photosystem I”, “carbon fixation”, and “circadian rhythm”. The DEGs in C4 were mainly related to “solute: cation symporter activity”, “water channel activity”, and “auxin-activated signaling pathway”. These results suggest that MT increased biomass by enhancing carbon fixation and photosynthesis in wheat seedlings under salt stress, and enhanced the response of wheat to salt stress by increasing the auxin-activated signaling pathway (Figure 6).

### 2.7. Transcription Factor and Co-Expression Network Analysis in S and SM Treatments

**Figure 7:**
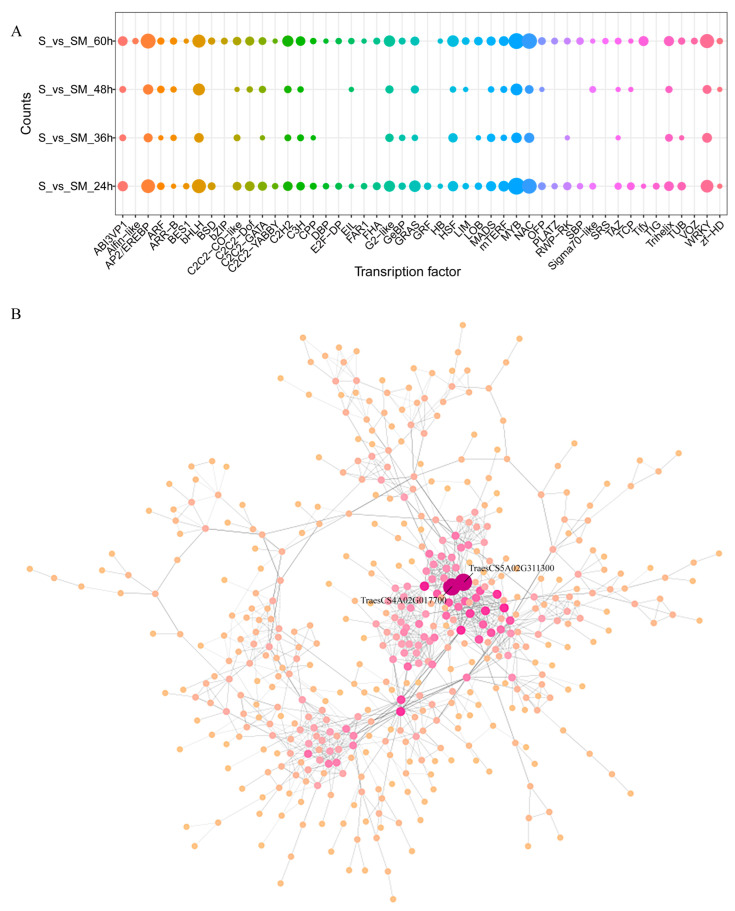
Transcription factor and co-expression network analysis in S and SM treatments. (**A**) Classifications of TFs in the SM-vs-S comparisons at different times; (**B**) Co-expression network of DEGs.

To investigate the regulation of transcription factors in wheat seedlings by MT under salt stress, the statistical analysis of transcription factors and network analysis were performed. A larger number of transcription factors (TFs) genes in 48 TF families were differentially regulated in wheat under salt stress (Figure 7A). The gene families with the highest number of differentially expressed TFs included the AP2/EREBP, bHLH, MYB, NAC, and WRKY families with 96, 97, 140, 110, and 81 DEGs, respectively (Figure 7A). Next, a co-expression network was created to identify key TFs associated with MT to improve salt tolerance in seedlings. Notably, *TraesCS4A02G017700* (*CDF1*) and *TraesCS5A02G311300* (*CBF14*) were at the center of the network and may be key TFs encoding genes induced by MT to regulate salt tolerance in wheat seedlings (Figure 7B).

### 2.8. Effect of Exogenous MT on LHC

**Figure 8:**
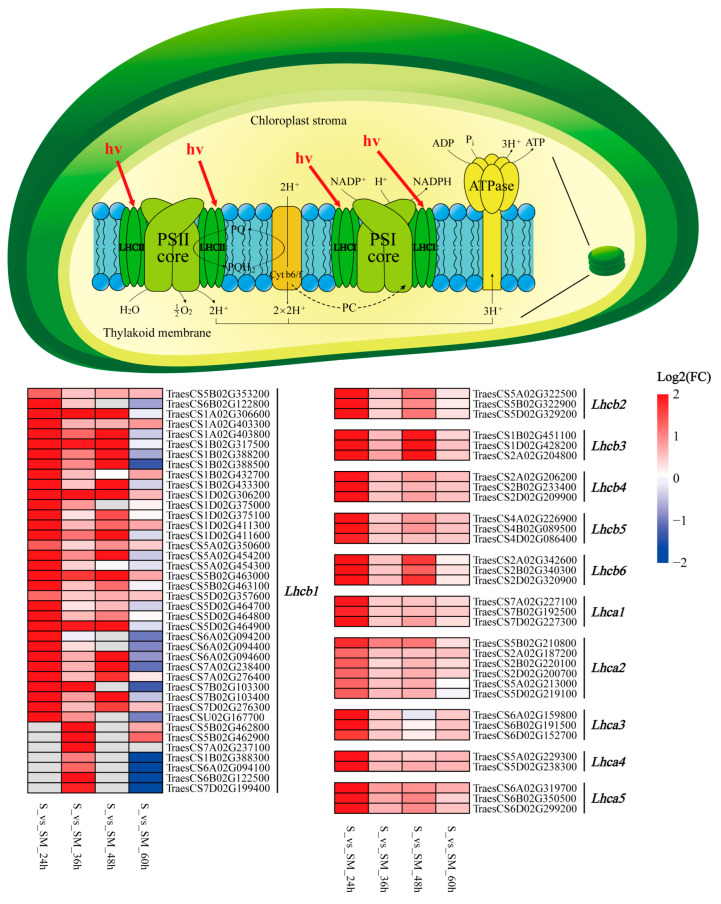
Photosynthesis light reaction process. PSⅠ: Photosystem Ⅰ; PSⅡ: Photosystem Ⅱ; LHCⅠ: Light-harvesting chlorophyll protein complex Ⅰ; LHCⅡ: Light-harvesting chlorophyl protein complex Ⅱ; PQ: Plastoquinone; PQH_2_: Plastoquinol-1; Cyt b6/f: Cytochrome b6/f complex; PC: Plastocyanin; ATPase: ATP Synthase.

Notably, the results of KEGG enrichment analysis show that photosynthetic antenna protein-related genes were significantly up-regulated by MT induction. The LHC, also known as the light-harvesting antenna complex, is responsible for collecting light energy for transmission to the photosynthetic reaction center, including LHCI and LHCII (Figure 8). In this study, 17 DEGs of five binding proteins in LHCI were found to be up-regulated at all times (Figure 8). A total of 55 DEGs encoding LHCII-related proteins, of which 40 Lhcb1 encoding genes, were up-regulated at 24, 36, and 48 h, while 15 DEGs encoding Lhcb2-6 were up-regulated at all times (Figure 8). The up-regulation of LHC-related genes indicates that MT improves photosynthesis in seedlings by increasing the leaves’ light-harvesting ability under salt stress.

### 2.9. Effect of Exogenous MT on Photosynthetic Pigments

**Figure 9:**
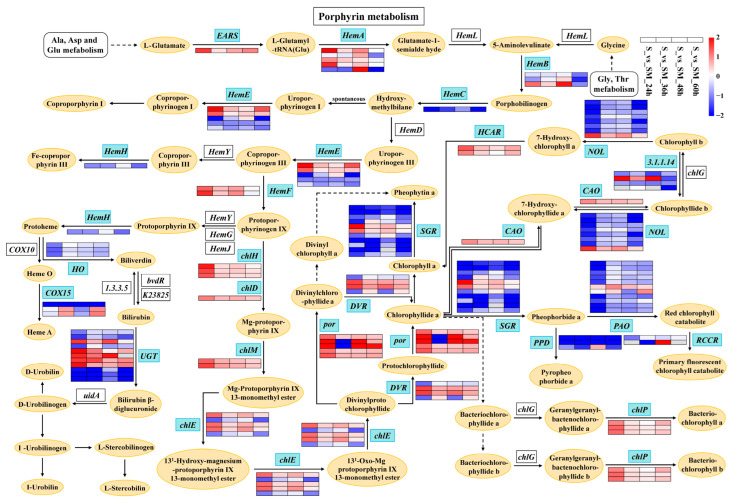
Porphyrin and chlorophyll metabolism pathway. *por*: protochlorophyllide reductase; *HemF*: coproporphyrinogen III oxidase; *HemE*: uroporphyrinogen decarboxylase; *HemB*: porphobilinogen synthase; *HemH*: protoporphyrin/coproporphyrin ferrochelatase; *EARS*: glutamyl-tRNA synthetase; *COX15*: heme a synthase; *HemA*: glutamyl-tRNA reductase; *chlH*: magnesium chelatase subunit H; *chlD*: magnesium chelatase subunit D; *chlM*: magnesium–protoporphyrin O-methyltransferase; *chlE*: magnesium–protoporphyrin IX monomethyl ester (oxidative) cyclase; *3.1.1.14*: chlorophyllase; *chlP*: geranylgeranyl diphosphate; *PAO*: pheophorbide a oxygenase; *UGT*: glucuronosyltransferase; *PPD*: pheophorbidase; *RCCR*: red chlorophyll catabolite reductase; *CAO*: chlorophyllide a oxygenase; *NOL*: chlorophyll(ide) b reductase; *HemC*: hydroxymethylbilane synthase; *HCAR*: 7-hydroxymethyl chlorophyll a reductase; *DVR*: divinyl chlorophyllide a 8-vinyl-reductase; *HO*: heme oxygenase (biliverdin-producing, ferredoxin); *SGR*: magnesium dechelatase.

Chlorophylls and carotenoids are involved in the uptake, transfer, and conversion of light energy in photosynthesis. A total of 100 DEGs in the “porphyrin and chlorophyll metabolism” pathway were regulated by MT (Figure 9 and Figure S2). Among them, *EARS*, *HemA*, and *HemF* were up-regulated by MT under salt stress (Figure 9 and Figure S2). MT induced the up-regulation of genes involved in chlorophyll biosynthesis, such as *chlD*, *chlM*, *por*, *DVR*, *HCAR*, and *chlP* (Figure 9 and Figure S2). However, MT down-regulated the expression of *HemC*, *HemH*, *HO*, *SGR*, *NOL*, *PAO*, and *PPD* in seedlings under salt stress (Figure 9 and Figure S2). These results indicate that MT improved chlorophyll biosynthesis and inhibited chlorophyll catabolism in seedlings.

**Figure 10:**
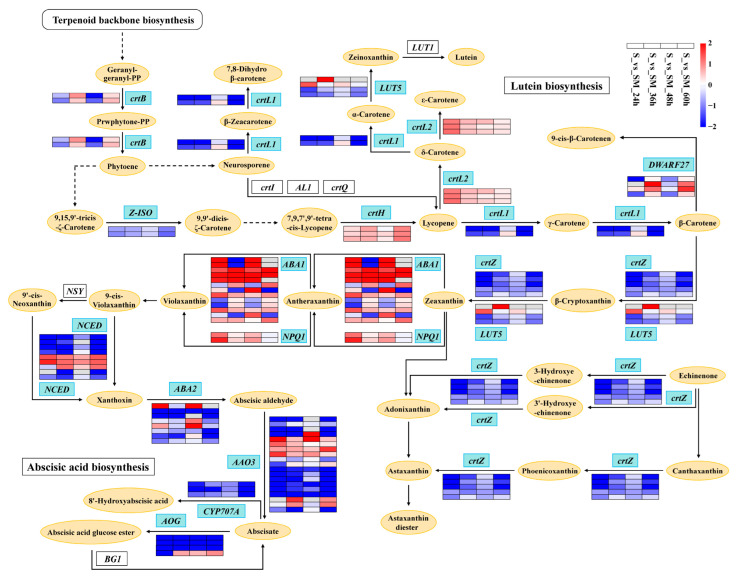
Carotenoid biosynthesis pathway. *crtB*: 15-cis-phytoene synthas; *crtL1*: lycopene beta-cyclase; *crtL2*: lycopene epsilon-cyclase; *crtH*: prolycopene isomerase; *ABA1*: zeaxanthin epoxidase; *NPQ1*: violaxanthin de-epoxidase; *NCED*: 9-cis-epoxycarotenoid dioxygenase; *ABA2*: xanthoxin dehydrogenase; *AAO3*: abscisic-aldehyde oxidase; *CYP707A*: (+)-abscisic acid 8’-hydroxylase; *AOG*: abscisate beta-glucosyltransferase; *Z-ISO*: zeta-carotene isomerase; *crtZ*: beta-carotene 3-hydroxylase; *LUT5*: beta-ring hydroxylase; *DWARF27*: beta-carotene isomerase.

A total of 83 DEGs in the carotenoid biosynthesis pathway were identified (Figure 10 and Figure S3). Among them, *crtL2*, *crtH*, *ABA1*, and *NPQ1* were up-regulated by MT under salt stress, whereas *CYP707A*, *AOG*, *Z-ISO*, and *crtZ* were down-regulated (Figure 10 and Figure S3). In the lutein biosynthesis process, the synthesis of ε-carotene was significantly up-regulated, while the synthesis of the lutein was significantly inhibited (Figure 10).

## 3. Discussion

### 3.1. Exogenous MT Alleviates the Growth Inhibition of Wheat Seedlings under Salt Stress

It is well known that crop growth, development, and production are inhibited by abiotic stresses [47]. However, plants have evolved different physiological, biochemical, and morphological strategies to cope with salt stress [48]. Growth inhibition is the most evident characteristic of plants exposed to environmental stresses [49]. The most commonly reported function of MT is to promote plant growth under abiotic stresses [50]. In the present study, exogenous MT application alleviated the inhibition of wheat growth caused by salt stress and significantly increased the plant height and biomass of wheat under salt stress (Figure 1), which is consistent with the results of earlier studies on canola, wheat, and naked oat [51,52,53]. Under salt stress, plant water absorption is hindered due to the disruption of the osmotic potential balance in the root cells, resulting in a physiological drought in the plant [13]. Exogenous MT promoted the accumulation of osmoregulatory substances, such as proline and soluble sugar, which maintain the osmotic balance of cells (Figure 2C,D).

Ionic toxicity occurs when Na^+^ concentration reaches a threshold in plant cells, and in severe cases necrotic spots are formed in the leaves [54]. In the present study, the leaf tip wilt index of wheat under salt stress was 55.27%, whereas the leaf tip wilt index of wheat applied with exogenous MT under salt stress was 15.18% (Figure 1D), indicating that MT significantly alleviated the leaf damage caused by salt stress. Meanwhile, osmotic stress and ion toxicity induced by salt stress caused the excessive accumulation of ROS in the electron transport process, which caused oxidative damage to the cytoplasmic membrane [55,56]. MT, which can scavenge H_2_O_2_ directly [57], is an effective antioxidant [40]. Our study indicated that exogenous MT increased the activities of antioxidant enzymes and reduced the accumulation of ROS in wheat to reduce oxidative damage induced by salt stress (Figure 2). Zhang et al. also found that MT increased the activity of antioxidant enzymes and reduced the accumulation of ROS in cucumbers under water stress [39].

### 3.2. Exogenous MT Alleviates the Photosynthetic Inhibition Affected by Salt Stress

Photosynthesis is the main driver of plant growth and production [58]. Plant growth has been reported to be affected by salt stress-induced reduced photosynthesis [59]. The decrease in photosynthesis under salt stress may be related to stomatal limiting factors and non-stomatal limiting factors [60]. The salt stress-induced reduction in photosynthesis is caused by the disruption of the light-harvesting chlorophyll protein complex (LHC) and Photosystem Ⅱ (PSII), the inhibition of chlorophyll biosynthesis, and the repair mechanism of PSII, blockage of electron transport, reduction in enzyme activity and CO_2_ supply, and promotion of stomatal closure [61,62,63,64]. Our study further demonstrated the role of MT in maintaining photosynthesis under stressful environments. Compared to the MT-untreated group, exogenous MT increased photosynthetic parameters and chlorophyll content to improve the ability of photosynthesis in wheat under stress (Figure 3). Cui et al. reported that MT increased gs and Tr in wheat under drought stress [65], illustrating that MT may influence WUE by affecting gs. Under salt stress, the assimilation rate may be influenced by the stomatal limitation factor and non-stomatal limitation factor [66]. Stomatal limiting factors include a decrease in gs, which limits the entry of CO_2_ from the air into the mesophyll cells and affects the photosynthetic rate. Non-stomatal limiting factors may reduce photosynthesis by inhibiting carbon assimilation, including decreased LHC light energy uptake, reduced PS activity, and reduced enzyme activity required for carbon fixation, resulting in the underutilization of CO_2_ and increase in Ci. In this study, we demonstrated that exogenous MT has a regulatory effect on non-stomatal limiting factors, exhibiting a significant increase in Ci under low gs and a significant decrease after the application of exogenous MT under salt stress (Figure 3B). These results suggest that MT may have increased the photosynthetic rate by increasing the efficiency of carbon assimilation in photosynthesis. In the study of cold-stressed wheat seedlings, the authors considered that MT enhances photosynthetic rate through its protective effects on Rubisco expression and photosynthetic pigments, thus ensuring the normal growth of wheat [67]. Zhou et al. suggested that MT could alleviate the inhibition of photosynthetic electron transport and D1 repair cycle protein synthesis under salt stress, thereby enhancing the tolerance of photosynthetic activity to salt stress in tomatoes [66].

### 3.3. Exogenous MT Enhances the Light Harvesting of Photosynthesis

Photosynthesis plays an important role in plant growth and development by converting light energy into chemical energy through two stages: photoreaction and carbon fixation. During the photoreaction stage, the LHC, which binds to PSII, is mainly involved in light harvesting, transfer, and photoprotection (Figure 8) [68,69]. In this study, we found that the genes encoding Lhca (Lhca1, Lhca2, Lhca3, Lhca4, Lhca5) and Lhcb (Lhcb1, Lhcb2, Lhcb3, Lhcb4, Lhcb5, Lhcb6, Lhcb7) were abundantly up-regulated by MT in response to salt stress (Figure 8). Jiang et al. found that the expression of Lhcb1 was up-regulated under cold, heat, salt, and drought stress in Apium graveolens [70]. Under osmotic stress, MT was able to up-regulate the levels of D1, Lhcb5, and Lhcb6 proteins and promote the dephosphorylation of LHCII and D1 [71]. Light is the source of energy for photosynthesis, and when the rate of energy absorption exceeds the rate of electron transfer, it leads to photooxidative damage [72]. The previous study reported that Lhcb4 plays a decisive role in LHCII and cannot be replaced by other Lhc subunits, while deletion mutants of Lhcb4 are defective in photoprotection [73]. Meanwhile, Lhcb5 (CP26) was demonstrated to be involved in non-photochemical quenching (NPQ) related to photoprotection in PSII [74]. Furthermore, *Lhca1*, *Lhca2*, *Lhca3,* and *Lhca4* encode the four antenna proteins which are responsible for connecting PSI and LHCB [75]. Our results suggest that the application of exogenous MT may enhance photosynthesis by improving the capacity of light energy capture and photoprotection in wheat leaves under salt stress.

### 3.4. Exogenous MT Affects the Synthesis of Chlorophylls and Carotenoids

Chlorophylls and carotenoids are two types of photosynthetic pigments in plants. It has been shown that a part of the chlorophyll converts light energy into chemical energy at the photoreaction center, while the rest of the chlorophyll and carotenoids bind to the LHC for light energy capture and transfer [76,77,78]. MT can mitigate the reduction in photosynthetic pigments and improve plant growth under stressful conditions [44,71]. Similar conclusions were obtained from our study. MT significantly increased the total chlorophyll content of wheat leaves under salt stress (Figure 3F). The levels of the chlorophyll synthesis precursor L-Glutamyl-tRNA were elevated due to the significant up-regulation of *EARS* (Figure 9), and genes related to the carotenoid synthesis pathway were also regulated by MT (Figure 10). Jahan et al. found that MT significantly up-regulated the expression of *protochlorophyllide oxidoreductase* (*por*), *chlorophyll a oxygenase* (*CAO*) genes, and increased chlorophyll content, in agreement with our findings [45]. In addition, violaxanthin and neoxanthin in the carotenoid synthesis pathway are also precursors for the biosynthesis of the phytohormone ABA [79]. Carotenoids, as auxiliary pigments in photosynthesis, can direct photons not absorbed by chlorophyll molecules to the reaction centers of photosynthesis [80]. Carotenoids also play an important role in photoprotection by quenching free radical triplet-state chlorophyll and singlet oxygen, or by active NPQ, or heat dissipation, before oxidative damage occurs [80,81,82]. These results indicate that exogenous MT improves the light-harvesting ability and photoprotective capacity of seedlings by increasing the total chlorophyll content and regulating the chlorophyll and carotenoid synthesis and metabolism, thereby improving the salt tolerance of wheat seedlings.

## 4. Materials and Methods

### 4.1. Materials and Treatments

Wheat “Zhongmai886” (ZM886) was used in this study. MT was purchased from Bio Basic Inc. (BBI, Shanghai, China) and all other chemicals were purchased from Sinopharm Chemical Reagent Beijing Co., Ltd. (Beijing, China).

Wheat seeds were sown in plastic pots (11 cm × 9 cm) containing a mixture of grass peat and vermiculite (1:1.5). To investigate the response of wheat seedlings to melatonin under salt stress, wheat seedlings were divided into control group (CK, normal conditions) and salt stress group (S, treated with 300 mM NaCl) at V2 stage. On the same evening, half of the seedlings in each group were sprayed with 5 mL of 100 μM MT in each pot of wheat leaves (CKM and SM) and the other half of the seedlings were sprayed with the same mass of distilled water (CK and S) for 5 days. The NaCl and MT concentrations were chosen in a pre-experiment. Each group of treatments had three biological replicates. The experiment was carried out in the Wisdom Agriculture Experimental Greenhouse at Beijing University of Agriculture, where were maintained at 25/20 °C (day/night), 40% relative humidity, 600 μmol·m^2^·s^−1^ photosynthetically active radiation, and 12/12 h (light/dark) photoperiod.

### 4.2. Morphological Observation

Morphological indices were measured after 20 days of MT treatment in seedlings. The plant height, length of the first leaf (L_1_), length of the wilt and yellowed part of the first leaf (L_2_), and root length of wheat seedlings were measured with a straightedge, and fresh dry weight was measured with an analytical balance. Three replicates were used for each treatment.

Leaf tip wilt index (%) = L_2_/L_1_ × 100%

### 4.3. Measurement of Malondialdehyde Content and Relative Electrolyte Leakage Rate (REL)

Physiological indicators were measured after 15 days of MT treatment in seedlings. All measurements were performed in three biological replicates. The content of malondialdehyde (MDA) was determined according to the method described by Hodges [83] and Diao [84] with some modifications. A total of 0.4 g of seedling leaves or roots and 8 mL 10% trichloroacetic acid (TCA) were added into a pre-cooled mortar and ground; then, they were centrifuged at 25 ℃ and 4000× *g* for 10 min and the supernatant was the crude extract of MDA. A total of 2 mL of the centrifuged supernatant (2 mL of distilled water for the control) was mixed with 2 mL of 0.6% thiobarbituric acid (TBA) and the MDA absorption was spectrophotometrically measured at 450, 532, and 600 nm. Relative electrolyte leakage was determined according to the method described by Dionisio [85] and Zhang [39]. A total of 1 g of fresh wheat leaves or roots were cut into pieces and placed in the test tube with 10 mL of distilled deionized water. The extracts were incubated in a constant temperature water bath at 32 °C for 2 h and the electrical conductivity (EC1) of the extracts cooled to 25 °C was measured using an electrical conductivity meter (DDS-307A, YOKE Instrument, Shanghai, China). The samples were boiled for 20 min to release all electrolytes and cooled to 25 °C to measure the boiling conductivity (EC2). The value of distilled deionized water was also measured (EC3). The REL was expressed by the following formula:

REL = (EC1 − EC3)/(EC2 − EC3) ×100%.

### 4.4. Measurement of Proline and Soluble Sugars Contents

The proline content was determined according to the method of Ye [86]. Briefly, 1 g of fresh wheat leaves or roots was homogenized in 10 mL of 3% sulphosalicylic acid and extracted in a boiling water bath for 10 min. Then, 2 mL extract liquid was incubated in a boiling water bath for 30 min with 2 mL ice acetic acid and 2 mL acidic ninhydrin solution. After cooling, the extract was extracted with toluene and measured at 520 nm. The soluble sugar content was determined according to the method described by Shi [87], with some modifications. The samples were incubated in a boiling water bath for 30 min. The supernatant after centrifugation (25 °C, 5000× *g*, 10 min) was mixed with 0.25 mL anthrone and 2.5 mL concentrated sulfuric acid and incubated in a boiling water bath for 10 min. The absorbance at 630 nm was measured and the content was calculated according to the standard curve of the sucrose standard.

### 4.5. Measurement of Reactive Oxygen Species and Antioxidant Enzyme Activities

The contents of H_2_O_2_ and O_2_^−^ in leaves and roots were determined according to the methods of Zhang [88] and Ke et al. [89]. Pre-cooled acetone was used to extract the H_2_O_2_ in the sample and detected by monitoring the absorbance of the titanium peroxide complex at 412 nm. A total of 1 mL of 65 mM phosphate buffer (pH = 7.8) was added to 0.3 g of samples, centrifuged in a centrifuge (4 °C, 5000× *g*, 15 min), and the supernatant was the O_2_^−^ extraction. The O_2_^−^ content of the leaves was detected by the absorbance at 530 nm of the pink azo compound formed by the hydroxylamine reaction.

Histochemical staining for H_2_O_2_ and O_2_^−^ was conducted according to the methods of Thorsten [90] and Dunand et al. [91]. H_2_O_2_ staining was performed by immersing fresh wheat leaves in a solution containing 1 mg/mL 3,3′-diaminobenzidine (DAB, pH = 3.0) under light-proof conditions. O_2_^−^ staining was performed by immersing fresh wheat leaves in 100 mM Nitro blue tetrazolium (NBT, dissolved in 50 mM phosphate buffer, pH = 7.5) solution. In both cases, the leaves were shaken in a shaker for 4–5 h (37 °C, 80–100 r/min). The leaves were rinsed with ethanol/lactic acid/glycerol (3:1:1; *v*/*v*) for 15 min in a 90–95 °C water bath to remove chlorophyll and any unreacted dye for observation later.

A total of 0.2 g of sample was added in a centrifuge tube with 2 mL phosphate buffer (pH = 7.8), and then centrifuged in a centrifuge (4 ℃, 12,000× *g*, 20 min), and finally the supernatant was collected for analysis of enzyme activities. The enzymatic activity of superoxide dismutase (SOD) was measured by its inhibition of the photochemical reduction in nitroblue tetrazolium using the method of Stewart and Bewley [92]. Peroxidase (POD) activity was determined by converting guaiacol to tetra guaiacol and measuring at 470 nm using the method of Andrea [93]. Catalase (CAT) activity was measured by detecting the change in absorbance of H_2_O_2_ at 240 nm using the method of Patra [94]. Ascorbate peroxidase (APX) activity was determined according to the method described in Nakano [95] and combined with the method in Zhou [96]. A total of 0.3 g of the sample was added to 2 mL of 50 mM PBS (pH = 7.8), 0.2 mM EDTA, 2 mM AsA, and then centrifuged in a centrifuge (4 °C, 12,000× *g*, 20 min). APX activity was assessed by measuring the change in absorbance over time at 290 nm.

### 4.6. Measurement of Gas Exchange Parameters and Chlorophyll Content

A portable photosynthesizer (CIRAS-3, HANSHA SCIENCE AND TECHNOLOGY GROUP CO., LIMITED, China, HongKong) was used to measure gas exchange parameters, including assimilation rate (A, μmol CO_2_ m^−2^ s^−1^), internal CO_2_ (Ci, mmol mol^−1^), stomatal conductance (gs, mmol H_2_O m^−2^ s^−1^), transport rate (Tr, mmol H_2_O m^−2^ s^−1^), and water use efficiency (WUE, mmol CO_2_ mol^−1^ H_2_O). Measurements were performed in a greenhouse with room and leaf temperatures maintained at 25 °C, reference CO_2_ at 425 µmol mol^−1^, and relative humidity between 60 and 80%. Chlorophyll content was measured using a chlorophyll meter (SPAD-502 PLUS, KONICA MINOLTA, Japan). Each treatment was replicated three times and the middle of the second leaf of wheat was selected for measurement.

### 4.7. RNA-Seq Analysis and Quantitative Real-Time PCR Assays

Total RNA was isolated from wheat leaves treated with S and SM after 24, 36, 48, and 60 h by using the Plant Total RNA Kit (Beijing Zoman, Beijing, China) according to the manufacturer’s protocol. Three biological replicates were set up for each treatment group at different time points. Transcriptome sequencing was performed by the Beijing Genomics Institute (BGI, Beijing, China) based on the MGISEQ-2000 platform. Clean reads were compared to genomic sequences by Bowtie2 (v2.2.5, Ben Langmead, Baltimore, MD, USA) and then gene expression was calculated for each sample using RSEM (v1.2.8, Bo Li, Madison, WI, USA) [97]. Identification of differentially expressed genes (DEGs) between SM and S treatments was performed using DESeq2 (v1.34.0, Michael I Love, Boston, Germany) [98]. *Padj* ≤ 0.05 and |log_2_FoldChange| ≥ 1.0 were used as standards to recognize the DEGs. The clusterProfile (v4.2.2, Guangchuang Yu, Guangzhou, China) was used for GO (gene ontology) enrichment analysis and (Kyoto Encyclopedia of Genes and Genomes) KEGG pathway analysis. Gene expression patterns were clustered using Mfuzz (v2.54.0, Lokesh Kumar, Berlin, Germany) in R [99].

Quantitative Real-time PCR (qRT-PCR) was used to test the reliability of the transcriptome data. Specific primers of DEGs were designed using the online quantitative primer database (https://bioinfo.ut.ee/primer3-0.4.0/, accessed on 12 April 2023). The primer sequence information of *TaActin1* (internal reference gene) and six randomly selected DEGs are shown in Table S1. LightCycler 96 Instrument (F. Hoffmann-La Roche Ltd., Indianapolis, IN, USA) was applied for qRT-PCR analysis. The samples used for the experiments were consistent with RNA-seq, with three biological replicates per treatment. Relative expression was calculated by the 2^−∆∆CT^ method [100].

### 4.8. Statistical Analysis

One-way ANOVA was performed using IBM SPSS Statistics 22 software (v22.0, IBM Corp, Armonk, NY, USA) for the evaluation of significant differences, and the final results qre expressed as mean ± standard error, with *p* < 0.05 being statistically significant.

## 5. Conclusions

The results of this study show that MT could effectively alleviate the growth retardation of wheat seedlings induced by salt stress. In addition, MT can reduce the accumulation of reactive oxygen species induced by salt stress, increase the activity of antioxidant enzymes, and alleviate the oxidative damage induced by salt stress. In this study, we found that exogenous MT increased the assimilation rate and chlorophyll content of wheat seedlings under salt stress, and MT improved the salt tolerance of wheat by regulating the expression of genes of photosynthetic capacity, photoprotection, and antioxidant-related pathways. Taken together, the present study revealed the regulatory mechanism of exogenous MT in improving the photosynthetic capacity and salt tolerance of seedlings under salt stress through the genes related to light capture and photoprotection through the measurement of physiological and biochemical indices and transcriptome analysis, which will provide a theoretical basis for the cultivation of new salt-tolerant varieties in the future.

## Data Availability

The RNA-seq dataset in this study has been uploaded to SRA database in NCBI (BioProject ID: PRJNA1034758).

## Supplementary Materials

The following supporting information can be downloaded at: https://www.mdpi.com/article/10.3390/plants12233984/s1.
