# Supplementary material for: Melatonin-Mediated Enhancement of Photosynthetic Capacity and Photoprotection Improves Salt Tolerance in Wheat
Source: Plants (Basel). 2023 Nov 27;12(23):3984. doi: 10.3390/plants12233984 (PMC10708427; doi:10.3390/plants12233984)
Supplement: Supplementary file 1 [file plants-12-03984-s001.zip › plants-2727249-Supplementary Materials.pdf]

Table S1. Primer sequences for qRT-PCR experiments.

Gene ID	F-primer	R-primer
TaActin1	CACCGCCGAACGGGAAAT	AAGGACCTCAGGGCAACG
TraesCS2B02G509100	CACTTCCTCATCGTGGCCTA	GTGGCGGTATCCTCACCATT
TraesCS1D02G019100	TCCACCTTCTCCCATTCCCC	CGCGTAGTTCCTCCGGATTT
TraesCS3D02G513900	GCGGTACAAGGTACGGGAAT	CGGCGAAATGGGGTCTACAT
TraesCS2B02G428700	GGGGAGGCTTTCGGGTTTTA	GTCGACGAACTCACCCTCAA
TraesCS2B02G132500	ACTACCTGTCCCAGCTCGT	CATCTGCCGGTCTGACATGA
TraesCS4A02G394600	CTTCTATGACGGCGAGGACC	GTACTCTGCCACGACGATCC

Table S2. Quality statistics of filtered transcriptome Reads

Sample Name	Clean Reads	Clean Base	Read Length	Q20(%)	Q30(%)	GC(%)
S_24h_1	39,381,657	11,814,497,100	PE150	97.33	93.02	53.14
S_24h_2	40,094,431	12,028,329,300	PE150	97.28	92.88	53.48
S_24h_3	40,116,715	12,035,014,500	PE150	97.28	92.87	53.33
S_36h_1	40,079,471	12,023,851,300	PE150	96.76	91.81	53.44
S_36h_2	40,088,085	12,026,425,500	PE150	96.86	92.04	53.53
S_36h_3	40,078,768	12,023,630,400	PE150	96.81	91.93	54.33
S_48h_1	40,137,198	12,041,159,400	PE150	97.00	92.38	53.00
S_48h_2	40,159,573	12,047,871,900	PE150	96.71	91.67	52.54
S_48h_3	40,059,088	12,017,702,400	PE150	96.87	92.00	52.63
S_60h_1	40,206,922	12,062,076,600	PE150	96.94	92.14	54.03
S_60h_2	40,239,658	12,071,897,400	PE150	97.01	92.29	54.16
S_60h_3	40,209,368	12,062,810,400	PE150	97.01	92.30	53.92
SM_24h_1	40,218,401	12,065,220,300	PE150	96.86	92.03	52.90
SM_24h_2	40,062,310	12,018,693,000	PE150	96.68	91.57	52.52
SM_24h_3	40,172,120	12,053,736,000	PE150	97.07	92.52	52.62
SM_36h_1	40,203,351	12,061,005,300	PE150	96.91	92.16	54.60
SM_36h_2	40,127,584	12,038,275,200	PE150	96.53	91.26	53.81
SM_36h_3	40,116,211	12,034,863,300	PE150	96.74	91.75	54.58
SM_48h_1	40,250,430	12,075,129,300	PE150	96.83	91.96	53.39
SM_48h_2	40,118,993	12,035,697,900	PE150	96.79	91.85	52.89
SM_48h_3	40,253,498	12,016,049,400	PE150	96.75	91.75	52.33
SM_60h_1	40,262,733	12,078,819,900	PE150	97.08	92.49	54.10
SM_60h_2	40,090,027	12,027,088,100	PE150	96.97	92.21	54.17
SM_60h_3	40,187,896	12,056,368,800	PE150	96.97	92.17	53.85

Table S3. DEGs up-regulate the most enriched GO terms.

ID	Description	p.adjust	Count
GO:0009768	photosynthesis, light harvesting in photosystem I	1.44E-30	33
GO:0015977	carbon fixation	1.15E-17	38
GO:0009734	auxin-activated signaling pathway	1.05E-12	49
GO:0071365	cellular response to auxin stimulus	1.05E-12	49
GO:0015995	chlorophyll biosynthetic process	1.09E-12	24
GO:0030244	cellulose biosynthetic process	5.90E-09	36
GO:0009833	plant-type primary cell wall biogenesis	6.59E-09	20
GO:0005516	calmodulin binding	5.22E-08	47
GO:0005372	water transmembrane transporter activity	1.81E-07	14
GO:0005887	integral component of plasma membrane	2.09E-06	49
GO:0009735	response to cytokinin	5.76E-06	24
GO:0016731	oxidoreductase activity, acting on iron-sulfur proteins as donors, NAD or NADP as acceptor	1.05E-05	8
GO:0005986	sucrose biosynthetic process	0.000116613	7
GO:0010206	photosystem II repair	0.000696603	8
GO:0042542	response to hydrogen peroxide	0.01786588	10
GO:0009753	response to jasmonic acid	0.023541287	14
GO:0009736	cytokinin-activated signaling pathway	0.02953734	8
GO:0009651	response to salt stress	0.031003989	30
GO:0009863	salicylic acid mediated signaling pathway	0.035821609	5
GO:0009738	abscisic acid-activated signaling pathway	0.036499141	18

Table S4. DEGs down-regulate the most enriched GO terms.

ID	Description	p.adjust	Count
GO:0046395	carboxylic acid catabolic process	3.24E-42	121
GO:0008514	organic anion transmembrane transporter activity	2.22E-27	92
GO:0006970	response to osmotic stress	2.46E-27	103
GO:0015171	amino acid transmembrane transporter activity	2.82E-24	60
GO:0009063	cellular amino acid catabolic process	1.24E-22	76
GO:0034440	lipid oxidation	3.81E-20	37
GO:0009062	fatty acid catabolic process	3.72E-18	34
GO:0009414	response to water deprivation	1.00E-15	62
GO:0006813	potassium ion transport	8.27E-11	33
GO:0012501	programmed cell death	1.64E-09	22
GO:0015996	chlorophyll catabolic process	7.22E-09	14
GO:0042402	cellular biogenic amine catabolic process	1.14E-07	18
GO:0005262	calcium channel activity	0.000261	6
GO:0098719	sodium ion import across plasma membrane	0.000696603	8
GO:0044092	negative regulation of molecular function	0.01786588	10
GO:0098659	inorganic cation import across plasma membrane	0.023541287	14

GO:0010150	leaf senescence	0.02953734	8
GO:0009852	auxin catabolic process	0.031003989	30
GO:0009938	negative regulation of gibberellic acid mediated signaling pathway	0.035821609	5
GO:0030245	cellulose catabolic process	0.036499141	18

Table S5. DEGs up-regulate the most enriched KEGG terms.

ID	Description	p.adjust	Count
ko00196	Photosynthesis - antenna proteins	2.41E-64	68
ko00860	Porphyrin and chlorophyll metabolism	1.54E-10	45
ko00402	Benzoxazinoid biosynthesis	3.27E-09	29
ko00531	Glycosaminoglycan degradation	4.81E-06	24
ko00906	Carotenoid biosynthesis	0.000144	32
ko01040	Biosynthesis of unsaturated fatty acids	0.004811	21
ko00770	Pantothenate and CoA biosynthesis	0.02485	17
ko00740	Riboflavin metabolism	0.030295	21
ko00604	Glycosphingolipid biosynthesis - ganglio series	0.030295	12
ko00310	Lysine degradation	0.038896	23
ko00908	Zeatin biosynthesis	0.044733	20

Table S6. DEGs down-regulate the most enriched KEGG terms.

ID	Description	p.adjust	Count
ko00943	Isoflavonoid biosynthesis	1.20E-10	38
ko00515	Mannose type O-glycan biosynthesis	1.20E-10	29
ko00590	Arachidonic acid metabolism	1.39E-10	46
ko00860	Porphyrin and chlorophyll metabolism	2.79E-10	55
ko00514	Other types of O-glycan biosynthesis	1.67E-09	41
ko00906	Carotenoid biosynthesis	1.82E-08	51
ko00944	Flavone and flavonol biosynthesis	5.31E-08	34
ko00402	Benzoxazinoid biosynthesis	1.18E-07	32
ko00650	Butanoate metabolism	2.06E-06	29
ko00440	Phosphonate and phosphinate metabolism	5.32E-06	12
ko00903	Limonene and pinene degradation	0.000813	9
ko04122	Sulfur relay system	0.002451	11
ko01040	Biosynthesis of unsaturated fatty acids	0.002716	27
ko00430	Taurine and hypotaurine metabolism	0.002873	12
ko00410	beta-Alanine metabolism	0.002895	38
ko00310	Lysine degradation	0.011406	32
ko00770	Pantothenate and CoA biosynthesis	0.011406	22
ko00942	Anthocyanin biosynthesis	0.011406	10
ko00232	Caffeine metabolism	0.026369	8
ko00750	Vitamin B6 metabolism	0.03313	12

ko00660	C5-Branched dibasic acid metabolism	0.034995	8
ko00100	Steroid biosynthesis	0.039027	34
ko04136	Autophagy - other	0.044899	36

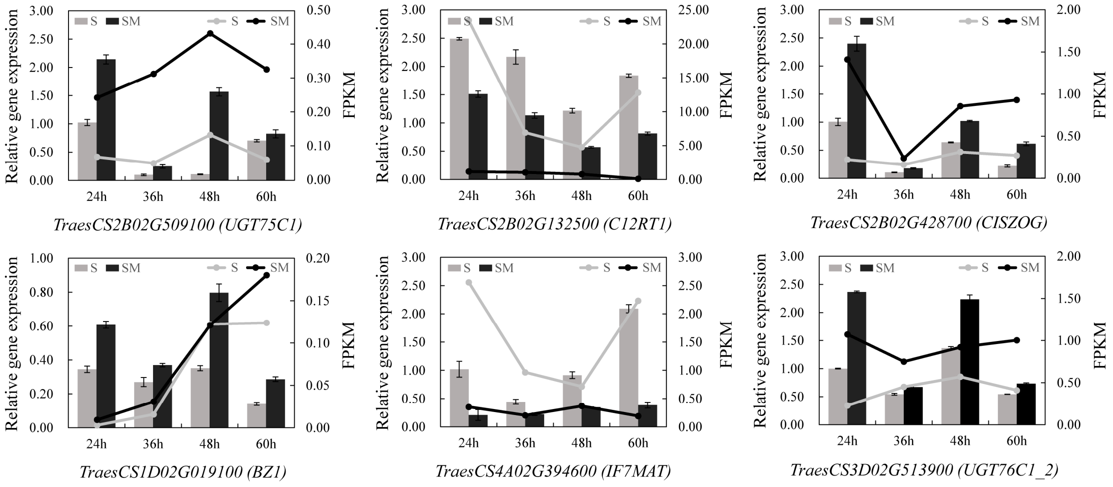

Figure S1. Effects of melatonin on gene expression under salt stress in wheat seeding by qRT-PCR.

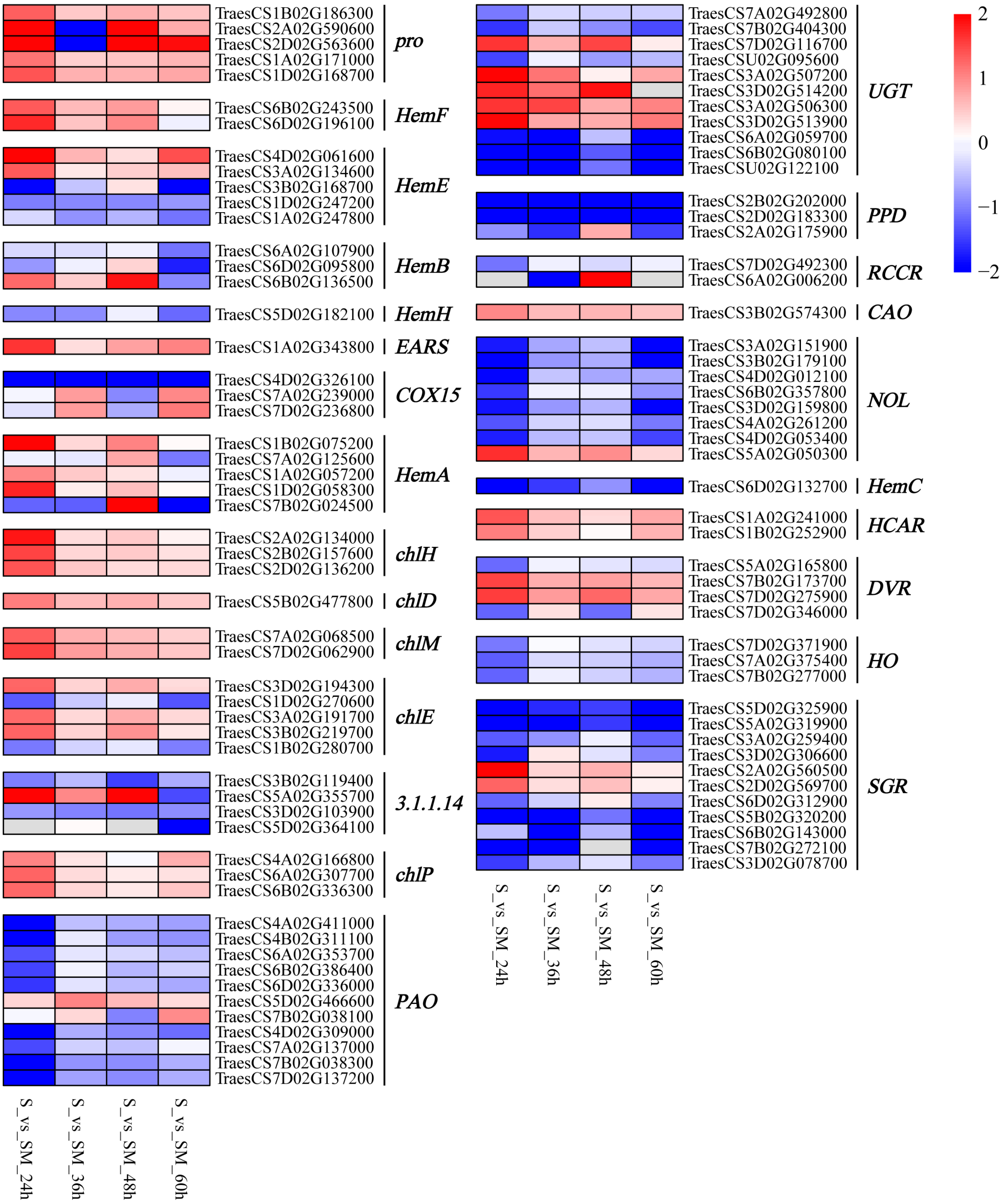

Figure S2. Expression profiles of DEGs associated with Porphyrin and chlorophyll metabolism.

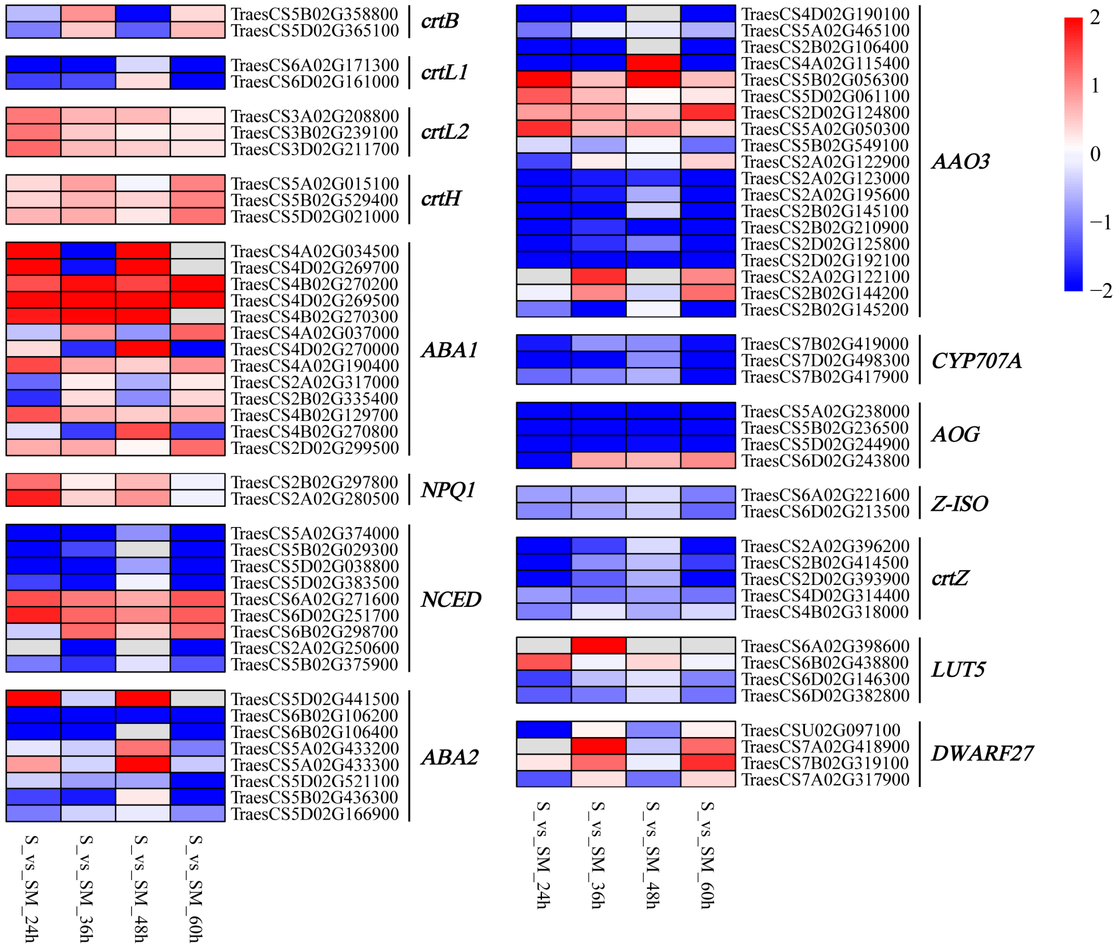

Figure S3. Expression profiles of DEGs associated with Carotenoid biosynthesis.
